# PBMC telomerase activity in depression and the response to electroconvulsive therapy

**DOI:** 10.1007/s00406-021-01294-4

**Published:** 2021-07-15

**Authors:** Karen M. Ryan, Martha Finnegan, Andrew Harkin, Declan M. McLoughlin

**Affiliations:** 1 Trinity College Institute of Neuroscience, Trinity College Dublin, Dublin 8, Ireland; 2 Department of Psychiatry, St. Patrick’s University Hospital, Trinity College Dublin, Dublin 8, Ireland; 3 School of Pharmacy and Pharmaceutical Sciences, Trinity College Dublin, Dublin 2, Ireland

**Keywords:** Telomerase, Depression, Electroconvulsive therapy, HAM-D24, PBMC

## Abstract

Telomerase, the DNA polymerase responsible for maintaining telomere length, has previously been implicated in depression and the response to antidepressant drugs. In this study, we aimed to compare telomerase activity in peripheral blood mononuclear cells between patients with severe depression recruited as part of the KEEP-WELL Trial (Ketamine for Depression Relapse Prevention Following ECT; NCT02414932) and age- and sex-matched healthy volunteers both at baseline/pre-ECT and at follow-up 1 month later for controls or in patients after a course of ECT. We found no differences in telomerase activity between patients with depression (*n* = 20) compared to healthy controls (*n* = 33) at baseline/pre-ECT, or between patients treated with ECT compared to controls at follow-up. In patients, telomerase activity was not associated with mood, as assessed by the 24-item Hamilton Rating Scale for Depression, or the duration of the current depressive episode. Additionally, we found no significant relationship between telomerase activity and exposure to recent or childhood adversity in either the patient or control groups. Overall, our results suggest that telomerase activity is not associated with depression, the therapeutic response to ECT, or exposure to adversity.

## Introduction

Psychiatric conditions such as major depressive disorder are suggested to be associated with accelerated cellular aging, indicated by shortened telomere length, amongst other factors [1]. The canonical function of the DNA polymerase telomerase, comprised telomerase reverse transcriptase (TERT) and a telomerase reverse transcriptase component (TERC), is to extend the 3′ ends of chromosomes by synthesizing telomeric repeats [2], termed “Okazaki fragments.” By adding to the lagging strand telomerase thereby maintains telomere length [3]. Telomere shortening leads to cellular senescence [4]. Thus, a deficiency of telomerase can lead to immune system and whole-body aging [4, 5], while enhancement of telomerase activity can prevent cellular senescence and promote longevity [4]. Telomerase also has several other functions. To this end, it is known to play an important role in angiogenesis, mitochondrial function, neurogenesis, reducing excitotoxicity, and apoptosis [3]. Studies have shown that increases in telomerase activity are negatively associated with levels of chronic stress, anxiety, dietary fat intake, cortisol, and glucose [6]. Unlike measuring changes in telomere length, which can take months to years to manifest, changes in telomerase activity can be measured over short periods of time owing to its dynamic nature [6].

Oxidative stress and inflammation, both of which have been reported to play a role in depression [7, 8], have been found to induce changes in telomerase activity [9–13]. While telomerase is less studied in psychiatric illness than telomere length, several preclinical and clinical studies have suggested a role for it in depression [14–20]. For instance, in the chronic mild stress rodent model of depression, telomerase activity was shown to be reduced in the hippocampus [14] and liver [17], while hippocampal telomerase activity was also found to be decreased in the Flinders Sensitive Line rat genetic model of depression [16]. In contrast, 12 weeks of chronic unpredictable stress exposure in rats was shown by others to increase telomerase activity in the blood [15]. Notably, these findings have been replicated in human studies of stress, with reports on individuals under chronic psychological stress showing reduced peripheral blood mononuclear cell (PBMC) telomerase activity, while a study of acute stress detailed increased telomerase activity in PBMCs (see 21 for review). Importantly, in rodents, the effects of stress on telomerase activity could be reversed by antidepressant treatment with the selective serotonin reuptake inhibitor (SSRI) fluoxetine [14], the tricyclic antidepressant desipramine [17], or the mood stabilizer lithium [16], and these effects appear to be related to telomerase-induced changes in neurogenesis [14].

Only a handful of previous studies have assessed telomerase activity in patients with depression, with conflicting results. In this regard, three studies with small sample sizes have shown telomerase activity to be increased in PBMCs from un-medicated patients with depression (*n* = 20–25) compared to healthy controls (*n* = 18–20) [18, 19, 22], while results from a fourth well-powered larger study (*n* = 166 per group) showed no significant difference in telomerase activity between un-medicated patients with depression compared to controls [20]. There has also been conflicting evidence regarding the relationship between telomerase activity and depression severity [18, 20]. In addition, Chen et al. [22] showed that telomerase activity was significantly increased in patients with depression compared to healthy controls and that in patients, but not in controls, greater exposure to adverse experiences during childhood was related to increased telomerase activity. However, Simon et al. [20] found no such relationship. Of note, a study by Wolkowitz et al. [18] showed that pre-treatment telomerase activity indicated a superior response to antidepressant treatment with the SSRI sertraline, with responders having significantly lower baseline telomerase than non-responders.

Electroconvulsive therapy (ECT) remains the most acutely effective treatment for severe, often life-threatening episodes of depression [23, 24]. However, its mechanism of action remains unclear [25]. To our knowledge, no study has yet examined telomerase activity in patients with depression undergoing treatment with ECT. Thus, here we examined telomerase activity in PBMCs collected from depressed patients pre- and post-treatment with ECT and from healthy controls at two time-points to approximate a course of ECT. We hypothesized that (1) telomerase activity would be lower in patients with depression compared to healthy controls and (2) telomerase activity would normalize in response to treatment with a course of ECT. We also explored whether there was any relationship between telomerase activity and depression severity or exposure to recent or early life adversity.

## Methods

### Participants

Ethical approval for this study was granted by the Research Ethics Committee of St James’s and Tallaght Hospitals (ref: 2014-08-19), and the study was performed in accordance with the Declaration of Helsinki [26]. All participants provided written informed consent.

All patients with depression were recruited as part of the KEEP-WELL Trial (Ketamine for Depression Relapse Prevention Following ECT; NCT02414932) in St. Patrick’s Mental Health Services, Ireland, between April 2015 and April 2017 [27]. The Maudsley Staging Method for Treatment Resistance in Depression was used to assess antidepressant treatment resistance, with scores of 3‒6 indicating mild resistance, 7‒10 indicating moderate resistance, and 11‒15 indicating severe resistance [29]. Patients recruited to the first phase of this trial underwent open-label ECT for the treatment of a major depressive episode. Brief-pulse ECT (1.0 ms pulse width; current amplitude 800 mA; maximum 1200 mC) was administered twice-weekly with hand-held electrodes, using a previously described stimulus dosing protocol [28]. Bitemporal ECT was administered at 1.5× threshold and right unilateral ECT (d'Elia placement) was given at seizure threshold. Methohexital (0.75–1.0 mg/kg) and succinylcholine (0.5–1.0 mg/kg) were used for anesthesia and muscle relaxation, respectively. Patients were maintained on their prescribed medications for the trial duration. To reflect real-world practice, the duration of the treatment course was determined by patients in consultation with their treating clinicians.

Participants met the following inclusion criteria: ≥ 18 years referred for ECT, unipolar major depressive disorder (DSM-IV), 24-item Hamilton Rating Scale for Depression (HAM-D24) score ≥ 21, and able to provide valid informed consent. Exclusion criteria were: any condition rendering the patient medically unfit for ECT or general anesthesia, active suicidal intent, dementia, intellectual disability, or Mini Mental State Examination (MMSE) score < 24, lifetime history of bipolar affective disorder, current history of post-traumatic stress disorder or any other Axis I diagnosis (DSM-IV), ECT in the 6 months prior to recruitment, alcohol dependence or substance misuse in the 6 months prior to recruitment, pregnancy or breast-feeding, residing in a nursing home, prisoner, diagnosis of a terminal illness, inability or refusal to provide valid informed consent.

Healthy controls were recruited through local newspaper and social media advertisements, and assessments were carried out one month apart.

### Clinical and demographic information

Clinical and demographic data were documented for both patients with depression and controls. Depression severity and response to ECT were assessed using the HAM-D24. Response to ECT was defined as ≥ 60% decrease from baseline HAM-D24 and a score ≤ 16 on two consecutive weekly ratings. Remission was defined as a ≥ 60% decrease from baseline HAM-D24 and a score ≤ 10 on two consecutive weekly ratings. The Childhood and Recent Trauma Events Questionnaire (CRTEQ) [30] was used to assess experiences of childhood trauma (CRTEQ-C; prior to the age of 17) as well as exposure to recent traumatic events (CRTEQ-R; within the past three years). The CRTEQ requests “yes/no” answers to questions about the occurrence of specific types of trauma experienced in childhood (before the age of seventeen) and recently (within the past three years). In the CRTEQ-C the categories of trauma assessed are bereavement, violence, traumatic sexual event, parental separation, serious illness or injury, and “other trauma,” while in the CRTEQ-R, the categories of trauma assessed are bereavement, spousal separation, violence, traumatic sexual event, serious illness or injury, change in work role, and “other trauma.” For each trauma type in the CRTEQ, respondents provide a score (using a 7-point scale, where 1 = not at all traumatic, 4 = somewhat traumatic, 7 = extremely traumatic) of their current perception of the severity of the trauma that they experienced in the past, which can be aggregated into a total trauma score for childhood or recent trauma.

### Blood sample collection

Fasting blood samples were taken in the morning between 07.30 and 11.30 am. For patients, blood samples were collected before the first ECT treatment and at 1–3 days post-treatment. Fasting control blood samples were taken in the morning on assessment days that were spaced 1 month apart. After the participant had rested quietly for 45 min, peripheral blood was collected into a sodium citrate Ficoll Cell Preparation Tube (CPT; BD, UK) and the tube was inverted 20–30 times. Following transport of the CPT tube from the hospital site to the laboratory (within 2 h), the tube was inverted 10 times prior to centrifugation at room temperature for 20 min at 1500×*g* with brakes off. The cell layer was collected and transferred to a 15 ml tube. Dulbecco’s Phosphate Buffered Saline (DPBS) was used to bring the volume to 10 ml and the tube was then capped and inverted 10 times. The tube was centrifuged at room temperature for 15 min at 300×*g* with brakes on. The supernatant was aspirated, and the cell pellet was re-suspended by tapping the tube. DPBS was added to bring the volume to 10 ml and the tube was capped and inverted 10 times. The tube was centrifuged at room temperature for 10 min at 300×*g* with brakes on. The supernatant was aspirated, and the cell pellet was re-suspended in 2 ml DPBS and a cell count was performed using a Sysmex KX-21 analyzer (Sysmex America Inc., USA). A volume of the cell sample containing 0.5 million cells was transferred to a micro-centrifuge tube and the tube was centrifuged at 7000 × rpm at 4 °C. The supernatant was aspirated, and the cell pellet was re-suspended in 100 µl of TRAPeze 1 × CHAPS lysis buffer (Merck Millipore, UK). The tube was left to sit on ice for 30 min and centrifuged at 14,000 × rpm for 20 min at 4 °C. The sample was aliquoted into cryogenic vials and aliquots were stored under liquid nitrogen until the telomerase assay was performed.

### Telomerase assay

Telomerase activity was determined using the TeloTAGGG Telomerase PCR ELISA kit (Roche, USA) according to manufacturer’s instructions. This kit utilizes a non-radioactive photometric technique whereby telomerase present in the samples will add repetitive TTAGGG sequences to the 3′-end of a biotinylated synthetic primer, while in a second step, the elongated products are amplified by PCR. The PCR amplicons are then detected using ELISA. A negative control was established for each cell extract sample to be tested by heating a 5 µl aliquot to 85 °C for 10 min prior to the assay to inactivate telomerase. Thus, each assay contained a positive extract control (from immortalized telomerase-expressing 293 cells, with telomerase intact), a negative extract control (from immortalized telomerase-expressing 293 cells, with telomerase heat inactivated as above), cell extract samples to be tested, and their heat inactivated negative controls. Baseline/pre-ECT and follow-up/post-ECT samples were run in the same batch to eliminate any differences caused by procedural variations. For step 1 of the assay, i.e., the telomeric repeat amplification protocol (TRAP) reaction, 25 µl Reaction mixture was added per tube followed by 3 µl cell extract/control, and 22 µl DNase-free water (Sigma-Aldrich, Ireland), and tubes were then transferred to a thermal cycler where the primer elongation/amplification protocol was as follows: primer elongation at 25 °C for 30 min, telomerase-inactivation at 94 °C for 5 min, 30 cycles of denaturation at 94 °C for 30 s, annealing at 50 °C for 30 s, and polymerization at 72 °C for 90 s, followed by 72 °C for 10 min, and hold at 4 °C. Step 2 of the assay, i.e., the hybridization and ELISA procedure, was performed per manufacturer’s instructions with the TMB (3,3′,5,5′-tetramethylbenzidine) reaction stopped after exactly 5 min. The absorbance was read at 450 nm with a reference wavelength of 630 nm. Telomerase activity was determined by subtracting the optical density readings of the heat inactivated negative controls from those of the cell extracts. Samples were deemed telomerase-positive where the difference in absorbance was greater than 0.2 A_450nm_–A_630nm_ units.

### Statistical analysis

To account for potential variation in telomerase assay batches, telomerase data from each batch were converted to z-scores after adjustment for slight variations in protein concentration across samples. Data were tested for normality using the Shapiro–Wilk test and Q-Q plots. Statistical analyses were performed for between-group comparisons of demographic and clinical data using independent *t* tests, Mann–Whitney *U* tests, or Chi-square tests (*Χ*^2^), where appropriate. A general linear model, both unadjusted and adjusted for potential confounders, was used to determine differences between groups. We included age, sex, smoking, body mass index (BMI; kg/m^2^), level of educational attainment, and presence of diabetes or heart disease as potential confounders since links between these factors and telomerase activity have previously been suggested [20, 31– 35]. Time of blood sample collection was also included as a potential confounder in an exploratory analysis. Correlation analyses were carried out using Pearson’s r or Spearman’s ρ where appropriate to determine relationships between continuous variables. Demographic and clinical data are presented as mean ± standard deviation (SD) or number (%) per group where appropriate. Telomerase activity data are presented as mean z-score ± SD. All data were analyzed using SPSS version 26 (IBM Corporation, NY, USA).

Using G*Power for analysis [36], with a sample size of 29 patients and 37 controls and setting alpha at 0.05 (two-sided), we had 80% power to detect an effect size of 0.33, which is a medium and clinically relevant effect size.

## Results

### Demographic and clinical characteristics of participants

For this study, PBMCs were available from 29 medicated patients with depression recruited to the KEEP-WELL trial and 37 healthy controls. Of these, we excluded seven patients and four controls with an inflammatory condition (e.g., psoriasis, ulcerative colitis, sarcoidosis) and two patients with a neurological disorder (e.g., Parkinson’s disease). In total, 20 patients with depression and 33 healthy controls were included in the final statistical analyses. Table 1 shows the demographic and clinical characteristics for the participants. BMI and educational attainment differed significantly between groups (*p* < 0.01).

**Table 1:** Demographic and clinical characteristics of participants

	Controls (*n* = 33)	Depressed (*n* = 20)	Statistical test
Age, years	51.03 (15.61)	58.05 (15.40)	*t* = 1.60, *p* = 0.12
Sex, No. (%)			
Male	14 (42.42)	10 (50)	*χ*^2^ = 0.29, *p* = 0.59
Female	19 (57.56)	10 (50)	
BMI	24.76 (4.28)	29.09 (6.14)	U = 175, *p* = 0.009
Smokers, No. (%)	3 (9.09)	6 (18.18)	*χ*^2^ = 3.46, *p* = 0.063
Education, No. (%)			
Primary	0 (0)	1 (5)	*χ*^2^ = 20.25, *p* = 0.00004
Secondary	4 (12.12)	14 (70)	
Tertiary and Quaternary	27 (81.82)	5 (25)	
Psychotic depression, No. (%)		1 (5)	
Baseline/Pre-ECT HAM-D24	3.23 (2.57)	27.95 (6.44)	U < 0.00, *p* = 1.94 × 10^–9^
Follow-Up/Post-ECT HAM-D24	2.91 (1.81)	11.50 (10.07)	U = 108, *p* = 0.00004
Electrode placement, No. (%)			
Unilateral		10 (50)	
Bitemporal		10 (50)	
Number of ECT sessions		11.5 (2.12)	
Responders, No. (%)		12 (60)	
Remitters, No. (%)		11 (55)	
Psychotropic medications, No. (%) taking			
SSRI		5 (25)	
Non-SSRI		14 (70)	
Lithium		7 (35)	
Lamotrigine		1 (5)	
Antipsychotic		16 (80)	
Benzodiazepine		9 (45)	
Treatment Resistant, No. (%)^a^			
Mild		3 (15)	
Moderate		17 (85)	
Severe		0 (0)	

Data are presented as means with standard deviations (SD) or number (%) per group where appropriate

*BMI* body mass index, *ECT* electroconvulsive therapy, *HAM-D24* Hamilton depression rating scale, 24-item version, *SSRI* selective serotonin reuptake inhibitor

^a^Assessed using Maudsley Staging Method for Treatment Resistance in Depression

### Telomerase activity in patients with a major depressive episode compared to healthy controls

**Fig. 1:**
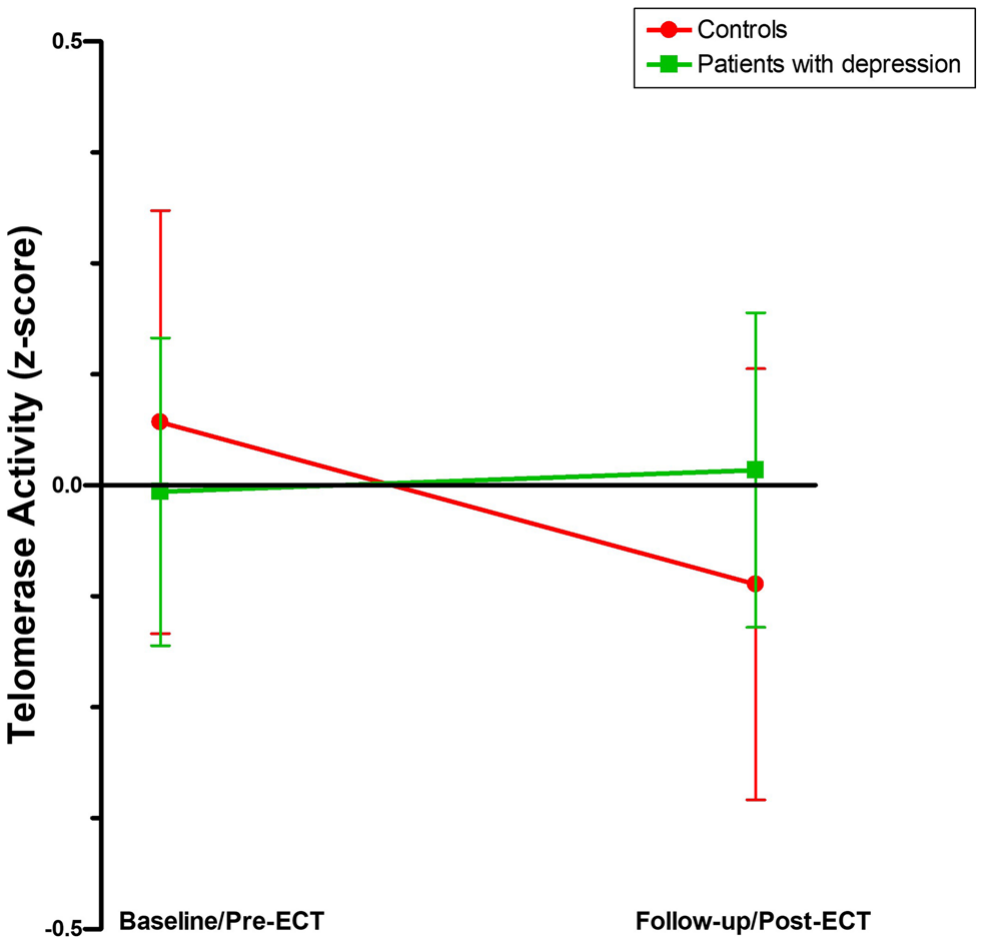
Telomerase activity in patients with depression compared to healthy controls. Data are shown as mean z-score ± standard deviation. Controls: *n* = 33; Patients with depression: *n* = 2

We first assessed baseline/pre-ECT telomerase activity in patients with depression compared to healthy controls (Fig. 1). Our unadjusted analyses show that telomerase activity did not differ significantly between patients (mean z-score ± SD: 0.071 ± 1.066) and controls (− 0.007 ± 0.996) (*F*_(1,51)_ = 0.073, *p* = 0.788). Adjusted analyses did not alter the results (*F*_(1,41)_ = 0.260, *p* = 0.613).

When we assessed the change in telomerase activity in patients with depression following a course of ECT compared to controls over time (− 0.111 ± 1.086 versus 0.017 ± 1.017, respectively; Fig. 1) our unadjusted analyses showed that there was no effect of time (*F*_(1,51)_ = 0.526, *p* = 0.472) or group (*F*_(1,51)_ = 0.009, *p* = 0.927) and no time × group effect (*F*_(1,51)_ = 0.900, *p* = 0.347). Adjusted analyses also showed no effect of time (*F*_(1,41)_ = 0.707, *p* = 0.405) or group (*F*_(1,41)_ = 0.817, *p* = 0.371) and no time × group effect (*F*_(1,41)_ = 0.927, *p* = 0.341). To account for potential effects of diurnal variation, adjusted analyses were also performed including the time at which the blood samples were collected, though this did not alter the results (data not shown).

An exploratory subgroup analysis showed that there was no significant difference in telomerase activity between female patients (*n* = 10) and controls (*n* = 19) (0.047 ± 1.012 versus − 0.142 ± 0.874, respectively; *F*_(1,27)_ = 0.274, *p* = 0.605) or between male patients (*n* = 10) and controls (*n* = 14) (0.096 ± 1.172 versus 0.175 ± 1.149, respectively; *F*_(1,22)_ = 0.028, *p* = 0.870).

Exploratory subgroup analyses were also conducted to determine if telomerase activity differed between ECT responders (*n* = 12) and non-responders (*n* = 8) or between ECT remitters (*n* = 11) and non-remitters (*n* = 9). There were no differences identified between these subgroups at baseline or over time (data not shown).

### Correlations between telomerase activity and mood

**Fig. 2:**
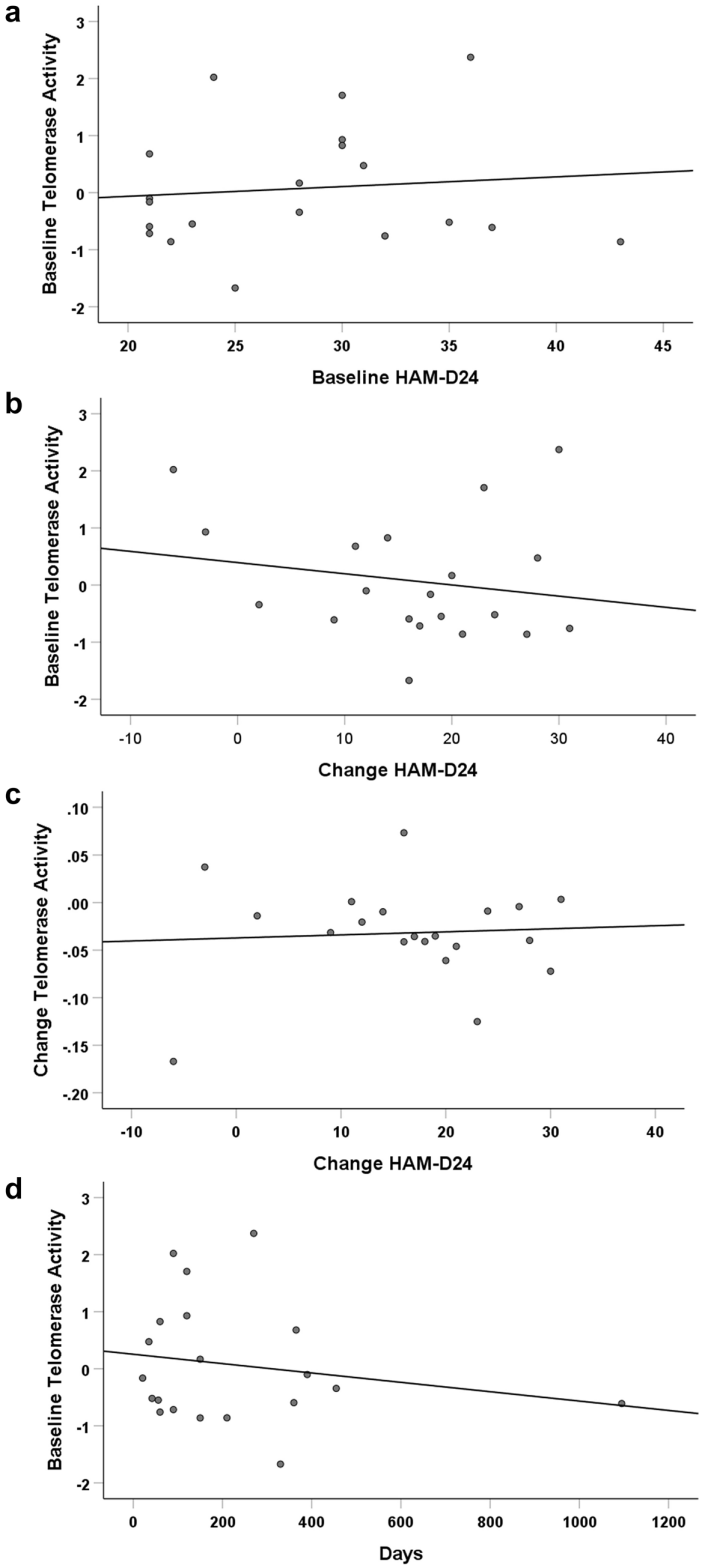
Correlations between telomerase activity and HAM-D24 scores in patients with depression. **a** Correlation between baseline telomerase activity and baseline HAM-D24 score. **b** Correlation between baseline telomerase activity and the change in HAM-D24 score post ECT. **c** Correlation between the change in telomerase activity and the change in HAM-D24 score post ECT. **d** Correlation between baseline telomerase activity and the duration of the current depressive episode in days. HAM-D24, 24-item Hamilton Rating Scale for Depression.

We subsequently examined if there was a relationship between telomerase activity and mood score, assessed using the HAM-D24. Baseline/pre-ECT HAM-D24 score did not correlate with baseline telomerase activity in patients with depression (*r* = 0.103, *p* = 0.666; Fig. 2a) and there was no relationship between baseline telomerase activity and the change in HAM-D24 score post-ECT (*r* = − 0.188, *p* = 0.428; Fig. 2b). There was also no relationship between the change in telomerase activity and the change in HAM-D24 score post-ECT (*r* = 0.045, *p* = 0.852; Fig. 2c). Moreover, there was no association between telomerase activity and the total duration of the current depressive episode (days) in the patient group (*ρ* = − 0.128, *p* = 0.591; Fig. 2d).

**Fig. 3:**
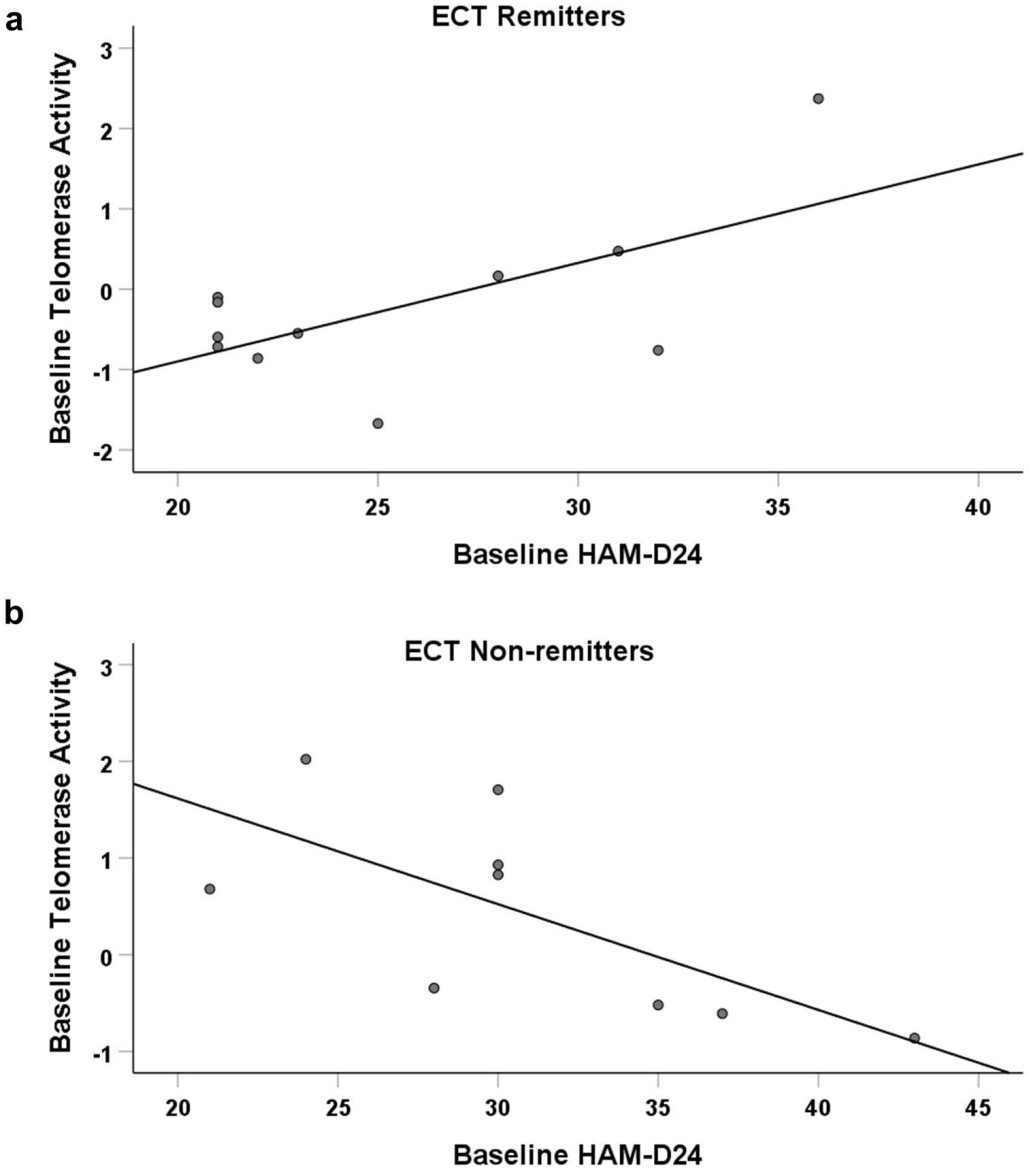
Correlations between baseline telomerase activity and baseline HAM-D24 score in ECT remitters and non-remitters. **a** Remitters. **b** Non-remitters. HAM-D24, 24-item Hamilton Rating Scale for Depression

A negative correlation was identified between baseline telomerase activity and baseline HAM-D24 (*r* = − 0.693, *p* = 0.038) in ECT non-remitters, indicating that a high baseline HAM-D24 score was associated with lower telomerase activity in this subgroup of patients (Fig. 3). However, it must be noted that the sample size for this subgroup was very small (*n* = 9). No other correlations were identified between telomerase activity and mood score in the responder/non-responder or remitter/non-remitter subgroups (data not shown).

### Telomerase activity and adverse experiences

CRTEQ data were available for 19/20 patients with depression and 29/33 controls. Of the patients with depression, 11 (58.9%) reported exposure to trauma on the CRTEQ-C, while 18 (54.5%) controls reported exposure to trauma on the CRTEQ-C. There was a trend towards significantly higher baseline telomerase activity in patients with (0.477 ± 1.202) compared to those without (− 0.370 ± 0.645) adverse childhood experiences on the CRTEQ-C (*F*_(1,17)_ = 3.250, *p* = 0.089). There was no difference in telomerase activity in controls with (-0.180 ± 0.821) and without (0.114 ± 1.229) adverse childhood experiences on the CRTEQ-C (*F*_(1,27)_ = 0.602, *p* = 0.445). Adjusting for potential cofounders revealed no significant difference between groups (patients: *F*_(1,10)_ = 3.094, *p* = 0.109; controls: *F*_(1,20)_ = 0.001, *p* = 0.978).

We also assessed telomerase activity in relation to exposure to recent traumatic experiences measured using the CRTEQ-R. Of the patients with depression, 16 (80%) reported experience of a recent trauma, while 23 (69.7%) controls reported exposure to a recent trauma. There was no difference in telomerase activity between patients with (*n* = 16; 0.235 ± 1.131) or without (*n* = 3; -0.491 ± 0.302) exposure to recent trauma on the CRTEQ-R (*F*_(1,17)_ = 1.167, *p* = 0.295). Adjusting for potential confounders did not alter this result (*F*_(1,10)_ = 0.032, *p* = 0.861). Baseline telomerase activity was significantly lower in controls with (*n* = 23; − 0.291 ± 0.831) compared to those without (*n* = 6; 0.784 ± 1.139) exposure to recent trauma on the CRTEQ-R (*F*_(1,27)_ = 6.839, *p* = 0.014). However, adjusting for potential confounders eliminated this difference (*F*_(1,20)_ = 2.712, *p* = 0.115).

In the group as a whole, there were no differences in telomerase activity with regard to the presence or absence of experiences of childhood or recent trauma (data not shown). Moreover, there was no correlation between telomerase activity and the total CRTEQ-C or CRTEQ-R scores in either the group as a whole or within the control or depressed groups individually (data not shown).

## Discussion

We found no difference in telomerase activity between medicated patients with depression compared to healthy controls, either at baseline/pre-ECT or follow-up/post-ECT. There was no association between telomerase activity and HAM-D24 score or the duration of the current depressive episode in the patient cohort. Our exploratory subgroup analyses found that telomerase activity did not differ between ECT responders and non-responders or remitters and non-remitters. With regard to the association between telomerase activity and exposure to trauma, we found a non-significant difference in telomerase activity in patients who had experienced adverse events during childhood compared to those who had not, though this did not survive adjustment for potential confounders. This trend was not evident in the control group, despite a similar percentage of people in each group having experienced adversity during childhood. While controls who had experienced recent traumatic events had significantly lower telomerase activity compared to those who had not in our unadjusted analysis, this did not survive adjustment for potential confounders.

As mentioned, only a handful of previous studies have assessed telomerase activity in patients with depression [18–20, 22]. Our results are in line with those from Simon et al. [20] who examined telomerase activity in a sample of well-characterized patients with depression (*n* = 166) (un-medicated at study entry) compared to age- and gender-matched healthy controls (*n* = 166) and showed that while telomerase activity was greater in patients compared to controls the difference was non-significant, even after controlling for covariates that differed between groups. Moreover, in line with our findings, the authors also reported that there was no association between telomerase activity and depression severity assessed using the Montgomery Åsberg Depression Rating Scale (MADRS) or the total depressive episode duration, again even after controlling for potential confounders including antidepressant use. Similar to our own findings, the authors of that study also showed that telomerase activity did not significantly differ according to age or gender, though male patients with depression had significantly greater telomerase activity than male controls, and female patients with depression had numerically lower telomerase activity than female controls. In contrast to both our own results and those of Simon et al. (2015), Wolkowitz et al. [18] found that baseline PBMC telomerase activity was significantly elevated in un-medicated patients with unipolar non-psychotic depression (*n* = 20) compared to healthy controls (*n* = 18), which the authors suggest represents a compensatory attempt to maintain telomere length in the face of cellular stress. Moreover, they found that telomerase activity correlated with self-reported depression and stress ratings at baseline in the entire study sample. Notably, in keeping with our own results, they found that there was no overall change in the average telomerase activity for the whole group from pre- to post-treatment with the SSRI sertraline. However, in contrast to our finding with respect to ECT, they showed that low baseline telomerase activity was positively correlated with the change in HAM-D17 score following sertraline treatment, and that those patients with relatively lower telomerase activity pre-treatment and a relatively greater increase in telomerase activity during treatment showed superior antidepressant responses to 8 weeks of sertraline. Chen et al. [22] also showed that telomerase activity was significantly increased in un-medicated patients with major depressive disorder (*n* = 20) compared to healthy age-, sex-, and ethnicity-matched controls (*n* = 20). In addition, a further study by Wolkowitz et al. [19] found that PBMC telomerase activity was higher in un-medicated patients with depression (*n* = 25) compared to controls (*n* = 18) and positively correlated with hippocampal volume in patients with depression but not in controls. This high telomerase activity in patients was again suggested to reflect compensatory mechanisms to enhance neurotrophic/neurogenesis effects in the brain under pathologic conditions.

Our unadjusted exploratory analyses show that there was a numerical difference in telomerase activity between patients with depression who had experienced adversity during childhood compared to those who had not, and between controls who had experienced a recent traumatic event compared to those who had not, though neither of these findings survived statistical adjustment for potential confounders. Our findings are, to some extent, in line with the results from the study by Chen et al. [22], which showed that telomerase activity was not associated with exposure to adversity in healthy controls but that adverse experiences during childhood were related to increased telomerase activity in patients with depression. Further studies are required to establish the exact links between exposure to stress/trauma and telomerase activity in both healthy and patient cohorts.

One reason why there might be a discrepancy in the results from our study and the study by Simon et al. [20] and those of Wolkowitz and colleagues [18, 19, 22] may be owing to the average age of the samples. The mean age of participants in the studies by Wolkowitz and colleagues was 35–38 years, while the mean age of our participants was 51–58 years and the mean age in the study by Simon et al. (2015) was 42 years. It has been reported that telomerase activity decreases with age, but that most of the decline occurs between birth and 40 years, after which time the levels remain relatively stable or non-existent [31]. In light of this, it has also been suggested that healthy young adults may more easily mount telomerase responses to protect their telomeres [15]. However, it is also possible, since our patients were receiving chronic pharmacotherapy, that long-term antidepressant exposure may have normalized telomerase activity back to control levels in our patient cohort, in line with the suggestion by Wolkowitz et al. [18] that the patients with the highest levels of telomerase activity in their study may already have achieved the maximum benefit from telomerase activation. A further difference between the studies conducted by Wolkowitz and colleagues and both ours and the study by Simon et al. [20] is the time at which blood samples were collected. Wolkowitz et al. had a strict blood collection time point of 8 am, while our study and that of Simon et al. (2015) were conducted under real-world conditions with bloods being collected over a wider time period.

Thus, there are several additional limitations to our study. First, the sample size of our study was small, in particular for subgroup analyses. Second, all of our patients were receiving pharmacotherapy treatment as usual prior to the initiation of the study and throughout the course of ECT treatment. Since others have shown telomerase activity to be altered by antidepressant treatment, it is possible that chronic antidepressant exposure normalized PBMC telomerase activity in our patient cohort prior to the study start. Third, we did not have central measures of telomerase activity, e.g., in cerebrospinal fluid. Therefore, while there were no differences in telomerase activity between controls and patients with depression at a peripheral level, this may not be the case at a central level since the relationship between central and peripheral telomerase activity is currently unknown. Moreover, while ECT had no effect on telomerase activity peripherally this does not rule out the possibility of it impacting on central telomerase activity. Fourth, this study was conducted under real-world conditions and so we were unable to tightly control the time point at which PBMCs were harvested. It has been shown that telomerase activity is subject to diurnal variation, with an increase in telomerase activity occurring between 8 am and 12 pm followed by a subsequent decrease Chen et al. [37]. That said, statistical adjustment for blood collection time point in our analyses revealed this had no impact on our results. One way to circumvent any potential variability in future studies may be through the measurement of maximal telomerase activity (mTAC), which assesses telomerase activity in cells in response to mitogen stimulation and has been shown to exhibit significant within-subject stability and between-subject variability and does not appear to be influenced by environmental factors or by the age of the subject [11]. Fifth, we did not have DNA available to perform telomere length analyses in this study. However, while large meta-analyses have shown shorter telomere length in patients with depression compared to controls [38–40], we identified no such difference in our previous study of telomere length in a large cohort of depressed patients referred for ECT compared to controls [41], and others have shown there is no relationship between telomere length and telomerase activity in depressed patients [18]. Notably, a previous study showed that while telomerase activity alone was not related to greater childhood adversity, those individuals who experienced greater exposure to adversity had a higher telomerase:telomere length ratio, which may be indicative of telomere endangerment [22]. Future studies should therefore assess both telomerase activity and telomere length in the same samples.

## Conclusion

We found no differences in telomerase activity in PBMCs from medicated patients with depression compared to controls, either at baseline/pre-ECT or at follow-up/post-ECT treatment. Thus, peripheral telomerase activity does not appear to be associated with the therapeutic response to ECT.

## Data Availability

Data available upon reasonable request.
